# Sewable soft shields for the γ-ray radiation

**DOI:** 10.1038/s41598-018-20411-3

**Published:** 2018-01-30

**Authors:** Seok Hyeon Gwon, Jeong-Hwan Oh, Minseok Kim, Sooseok Choi, Kyu Hwan Oh, Jeong-Yun Sun

**Affiliations:** 1 Department of Materials Science and Engineering, Seoul National University, Seoul, 08826 Republic of Korea; 2 Research Institute of Advanced Materials (RIAM), Seoul National University, Seoul, 08826 Republic of Korea; 3 Department of Nuclear and Energy Engineering, Jeju National University, Jeju, 63243 Republic of Korea

## Abstract

Soft shields are required to protect the human body during a radioactive accident. However, the modulus of most soft shields, such as HDPE and epoxy, is high, thereby making it difficult to process them in wearable forms like gloves and clothes. We synthesized a soft shield based on a hydrogel that is very compliant, stretchable, and biocompatible. The shields were fabricated by integrating γ-ray-shield particles into hydrogels with an interpenetrating network. The soft shields containing 3.33 M of PbO2 exhibited a high attenuation coefficient (0.284 cm^−1^) and were stretched to 400% without a rupture. Furthermore, the fabricated soft shield can be sewn without a fabric support due to its high energy-dispersion ability. A wearable arm shield for the γ-ray radiation was demonstrated using a direct sewing of the soft-shield materials.

## Introduction

In 2011, the Fukushima Daiichi nuclear disaster occurred due to the Great East Japan Earthquake and tsunami. Radioactive isotopes were released from reactor-containment vessels, and the Japanese government implemented an exclusion zone around the power plant^1^. The World Health Organization (WHO) released a report that predicted an increase of the risk regarding specific health effects for the populations living around the Fukushima nuclear-power plant^2^. Accordingly, the anxiety about radiation leaks and the interest in radiation shields increased.

The radiation that is emitted from radioactive materials can be classified as either α, β, γ, or neutron radiation according to the energy and the form of the radiated waves or particles^3^. The α and β rays with low-energy levels are easily blocked by an aluminum plate, while the γ-ray with a high-energy level can effectively be shielded by metals such as iron (Fe), tungsten (W), and lead (Pb) that comprise high atomic numbers and densities^4^. They decrease the transmission rate of the γ-ray through an interaction between the orbital electrons and the γ-ray^5^. Concrete compounds are generally used to shield the γ-ray, but because they are bulky and heavy, metal-concrete composites are used only for nonmobile structures (i.e., buildings)^6–8^. Polymer-matrix metal composites such as high-density polyethylene (HDPE) or epoxy composited with high-density metal particles have recently garnered attention as a γ-ray shield material^9,10^. These polymers are less efficient for shielding the γ-ray but are relatively light compared to metals and concrete compounds. However, HDPE and epoxy are naturally stiff, making them difficult to process for clothing manufacturing because of their high modulus^11,12^.

Hydrogels are very compliant materials that, like the human skin, have a Young’s-modulus range from 10 kPa to 10 Mpa^13–16^. However, hydrogels are rarely used for structural materials because most hydrogels are brittle^17,18^. Therefore, researchers have struggled to develop a mechanically tough hydrogel with the use of many strategies such as interpenetrating networks and nanocomposites^19–21^. Among them, the hydrogel with an interpenetrating network is composed of ionically and covalently crosslinked networks, which can be stretched to 20 times their initial length and have fracture energies of 9,000 J/m^2 ^^22^. Furthermore, despite the presence of a notch in the hydrogels, it can be stretched to 17 times their initial length due to the dissipation of the concentrated energy through the double network^22^. Also, hydrogels can be used as a neutron-shielding material because they contain ~90% water.

In this study, a wearable soft shield was fabricated from hydrogels that had been integrated with shield particles. To improve the wearability of the soft shield, the focus was the mechanical properties of the soft shield, especially the sewability.

## Experimental

### Gel fabrication

**Figure 1:**
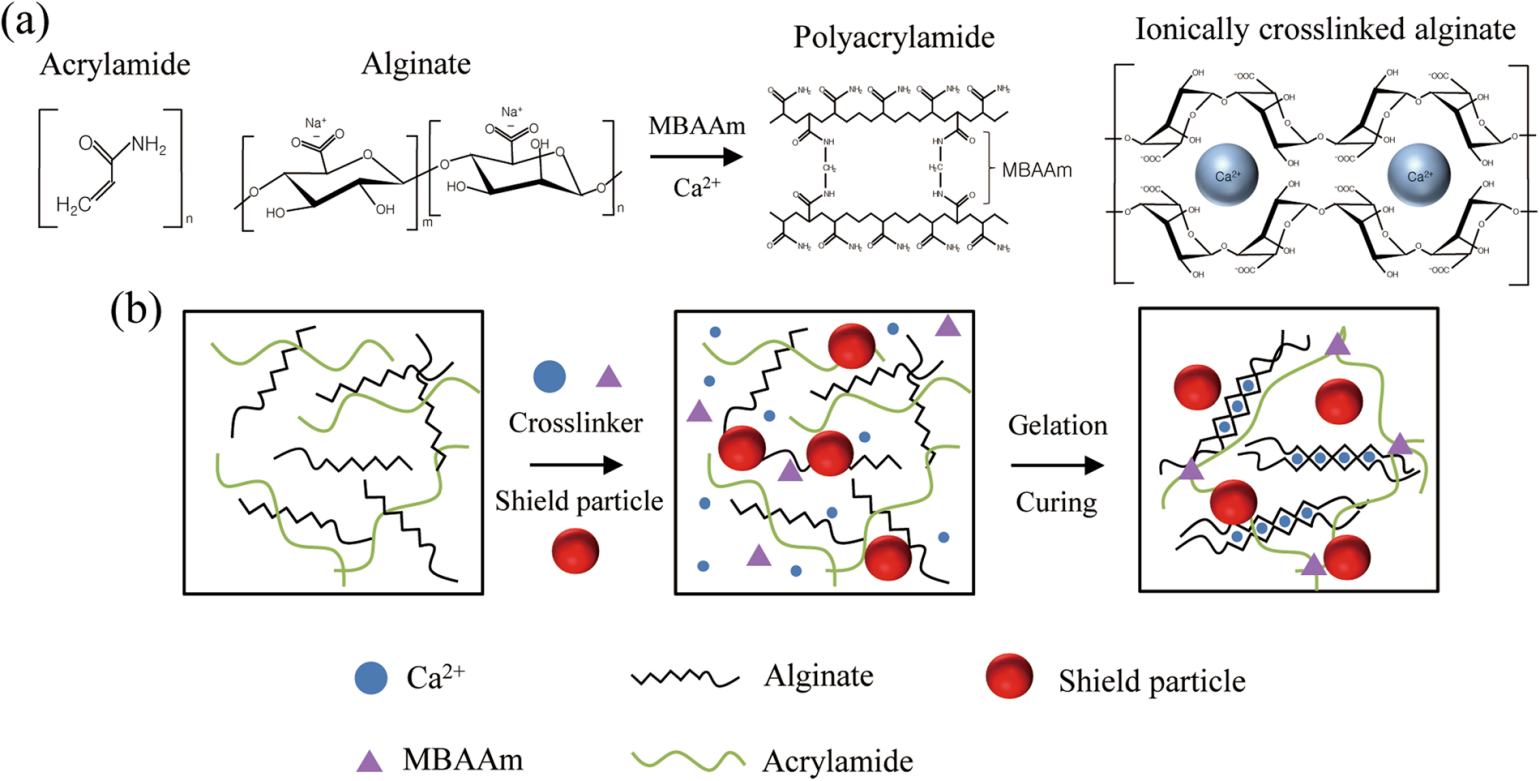
Synthesis procedures of soft shields for the γ-ray radiation. (**a**) Polyacrylamide was covalently crosslinked with N,N-methylenebisacrylamide (MBAAm), and alginate was ionically crosslinked with the Ca^2+^ cation. (**b**) The soft shield for the γ-ray radiation was synthesized by integrating the microshield particles into a highly stretchable and soft hydrogel matrix.

Here, the synthesis of a sewable soft shield for the γ-ray radiation was achieved through the integration of hydrogels and metal oxides, for which 1 g of sodium alginate and 8 g of acrylamide were dissolved in deionized water (86 wt% water contents) and stored at 4 °C for 3 days to obtain a homogeneous solution. N,N,N′,N′-tetramethylethylenediamine and 0.1 M N,N-methylenebisacrylamide (MBAAm) were added as a crosslinking accelerator for the polyacrylamide and a crosslinker for the polyacrylamide, respectively. A metal-oxide (Fe_2_O_3_, WO_3_, and PbO_2_) slurry was added into the alginate/acrylamide solution. Then, a solution comprising 0.2 M ammonium persulphate and 1.22 M calcium sulfate was added as a thermal initiator for the polyacrylamide and as an ionic crosslinker for the alginate. A uniformly distributed hydrogel solution was poured into a glass mold and cured with ultraviolet light (with 8 W of power and a 254-nm wavelength) for 1 hr before it was stabilized at room temperature for 2 days. The overall synthesis procedure of the soft shields is shown in Fig. 1.

## Results and Discussion

**Figure 2:**
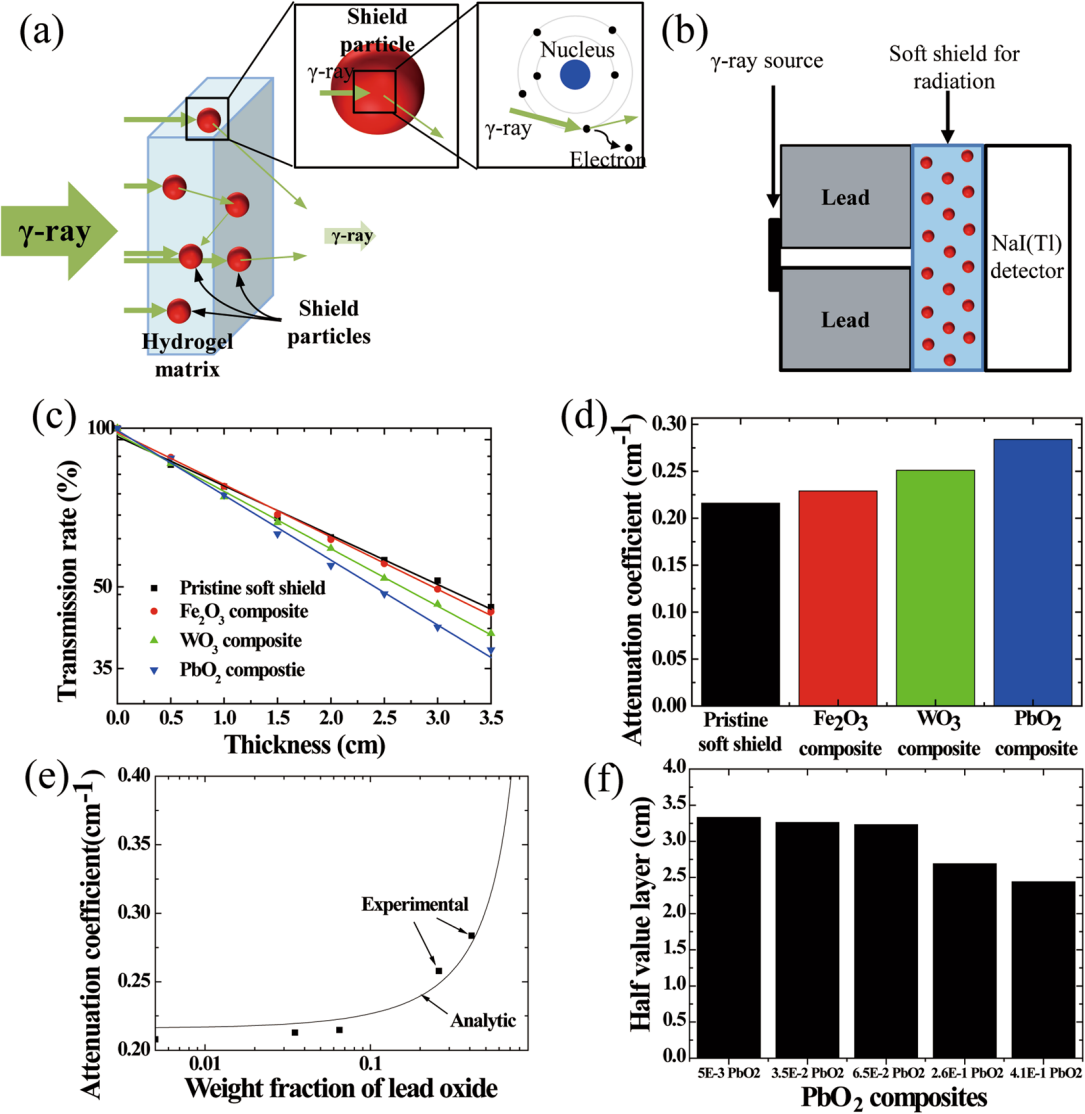
(**a**) The attenuation principle of the γ-ray radiation according to soft shields. The γ-ray was attenuated by interactions between electrons and shield particles. (**b**) A schematic illustration for an experimental measurement of the γ-ray transmission with a Cs-137 (0662 MeV) radiation source. (**c**) The transmission rates for the γ-ray radiation were investigated using the thickness of the soft shields. The shields contain 3.33 M of each shield particle. (**d**) The attenuation coefficients of the soft shields were evaluated from the transmission rates. (**e**) The calculated attenuation coefficient (solid line) and the comparison with the measurements (filled square data) for a lead oxide (PbO_2_) composite. (**f**) Variation of the half-value layer with the PbO_2_ content in the soft shields. 5E-3 PbO_2_, 0.005 weight-fraction PbO_2_ content; 3.5E-2 PbO_2_, 0.035 weight-fraction PbO_2_ content; 6.5E-2 PbO_2_, 0.065 weight-fraction PbO_2_ content; 2.6E-1 PbO_2_, 0.26 weight-fraction PbO_2_ content; and 4.1E-1 PbO_2_, 0.41 weight-fraction PbO_2_ content.

The principle of the soft-shield γ-ray attenuation is shown in Fig. 2(a). The shielding of the γ-ray radiation was primarily induced by an interaction between the electrons in the shield particles and the γ-ray^5,23^. The γ-ray interacted with an atom resulting in the ejection of an electron. The electron then received energy from the γ-ray, and this may induce the secondary ionization of the electron. During the passing of the γ-ray through the shielding material, the γ-ray intensity was decreased. To estimate the ability of the γ-ray shield, it is important to quantify the transmitted γ-ray. The transmission rate and the attenuation coefficient are shown by Equation (1), as follows:

1 $${Transmission}\,{rate}(T)=I/{I}_{{0}}={e}^{-\mu t},$$

where *I* is the postshielding intensity, *I*_*0*_ is the incident intensity, *μ* is an attenuation coefficient, and *t* is the shielding-material thickness^24^. The mass attenuation coefficient (*μ*_*m*_) of the compound is shown by Equation (2), as follows:

2 $${\mu }_{m}=\mu /\rho ={\sum }_{i}{w}_{i}{({\mu }_{m})}_{i}$$

where *ρ* is the shield-material density, and *w*_*i*_ and (*μ*_*m*_)_*i*_ are the weight fraction and the mass attenuation coefficient of the ith-constituent element, respectively. For the present work, metal oxides and hydrogel were used as the constituents.

To measure the transmission rate of the passing of the γ-ray through the shielding material, the device that is shown in Fig. 2(b) was customized for the present study. The radiation-source stand was designed with the inner, vertical, and horizontal diameters of 5 mm, 40 mm, and 25 mm, respectively. A γ-ray path from the source was sealed with a lead-metal shield to prevent the radiation scattering toward the detector. The soft shield was placed between the detector and the radiation-source stand. Then, the transmission rate of the soft shields was measured using the previously mentioned device. The radiation source of Cs-137 (0.662 MeV) was manufactured in November 2011, and the activity is 5 μCi. Various soft shields with a thickness of 5 mm were used in the transmission-rate measurements. During the measurements, 800 V of voltage were applied to the detector three times for 600 s each time.

Figure 2(c) shows a logarithmic-scale plot of the γ-ray-transmission rates as a function of the soft-shield thickness. The shields contain 3.33 M of each shield particle. The γ-ray-transmission rate was decreased by an interaction between the orbital electrons of the metal oxide and the γ-ray. As shown in Fig. 2(c), the transmission rate was linearly decreased as the soft-shield thickness was increased. In addition, the transmission rate of the soft shields containing lead oxide (PbO_2_) powder is much lower than that of the other soft shields containing tungsten trioxide (WO_3_) and ferric oxide (Fe_2_O_3_) powders, suggesting that as the atomic number of the metal-oxide powder was increased, the interaction probability between the γ-ray and the electron was increased proportionally. Therefore, the soft shield including heavy elements such as PbO_2_ powder is more effective than those containing WO_3_ and Fe_2_O_3_ powders; accordingly, the soft shield including WO_3_ powder is more effective than that including Fe_2_O_3_ powder.

To obtain the attenuation coefficients for the soft shields including the metal-oxide powders, a slope was fitted with a graph, as shown in Fig. 2(c). The attenuation coefficient of the soft shield for the Cs-137 γ-ray at 0.662 MeV was evaluated using Equation (1). Figure 2(d) shows the attenuation coefficients of the various soft shields. The attenuation coefficient of the pristine soft shield without any metal-oxide powder is 0.216 cm^−1^, which is higher than that of the soft shield with metal aluminum (0.2 cm^−1^)^25^. This result is attributed to the high mass attenuation coefficient and the suitable molecular structure of the water for the γ-ray-radiation shielding. In the cases of the soft shields including iron, tungsten, and lead oxides, the attenuation coefficients are 0.229, 0.251, and 0.284 cm^−1^, respectively. For the soft shields with a metal oxide containing a high atomic number, the attenuation coefficients of the soft shields were increased. Accordingly, the volume of the heavy-metal particles per unit volume is a primary factor of the radiation-shield ability. Pure metals (i.e., Fe, W, and Pb) will show more favorable attenuation abilities than metal oxides because they contain more metal atoms per unit volume. But metals are unstable in hydrogel because they will be gradually oxidized. Therefore, the investigation of the metal oxides is only regarding the stability of the shield.

Using Equation (2), the attenuation coefficient for the added amount of lead oxide is shown in Fig. 2(e). Analytic calculations were made with values of hydrogel (*ρ*_*hydrogel*_ = 1.13 g cm^−3^, *μ*_*hydrogel*_ = 0.216 cm^−1^) and bulk lead oxide (*ρ*_*Pbo*2_ = 9.38 g cm^−3^, *μ*_*Pbo*2_ = 1.020 cm^−1^). The density and attenuation-coefficient measurements of the hydrogel are presented in Fig. 2(d). The lead-oxide attenuation coefficient at 0.662 MeV was calculated using the XCOM (National Institute of Standards and Technology, U.S.A.) program. The theoretical attenuation coefficient of the lead oxide composites at 0.662 MeV was compared with the measured values in Fig. 2(e). The experimental values are in a sound agreement with the theoretical calculations. The attenuation coefficient for the lead oxide composite sharply increased with the increasing of the weight fraction.

The half-value layer (HVL)—it was necessary to reduce the incident intensity of the γ-ray by half using the thickness of the radiation-shielding material—can be calculated using Equation (1), as follows:

3 $$HVL=\frac{-ln0.5}{\mu }.$$

The effectiveness of the gamma-ray shielding is described in terms of the HVL of the PbO_2_ composites, as shown in Fig. 2(f). The lower the HVL value, the more effective the radiation material in terms of the thickness requirement. The increase of the PbO_2_ content in the soft shield decreased the HVL.

**Figure 3:**
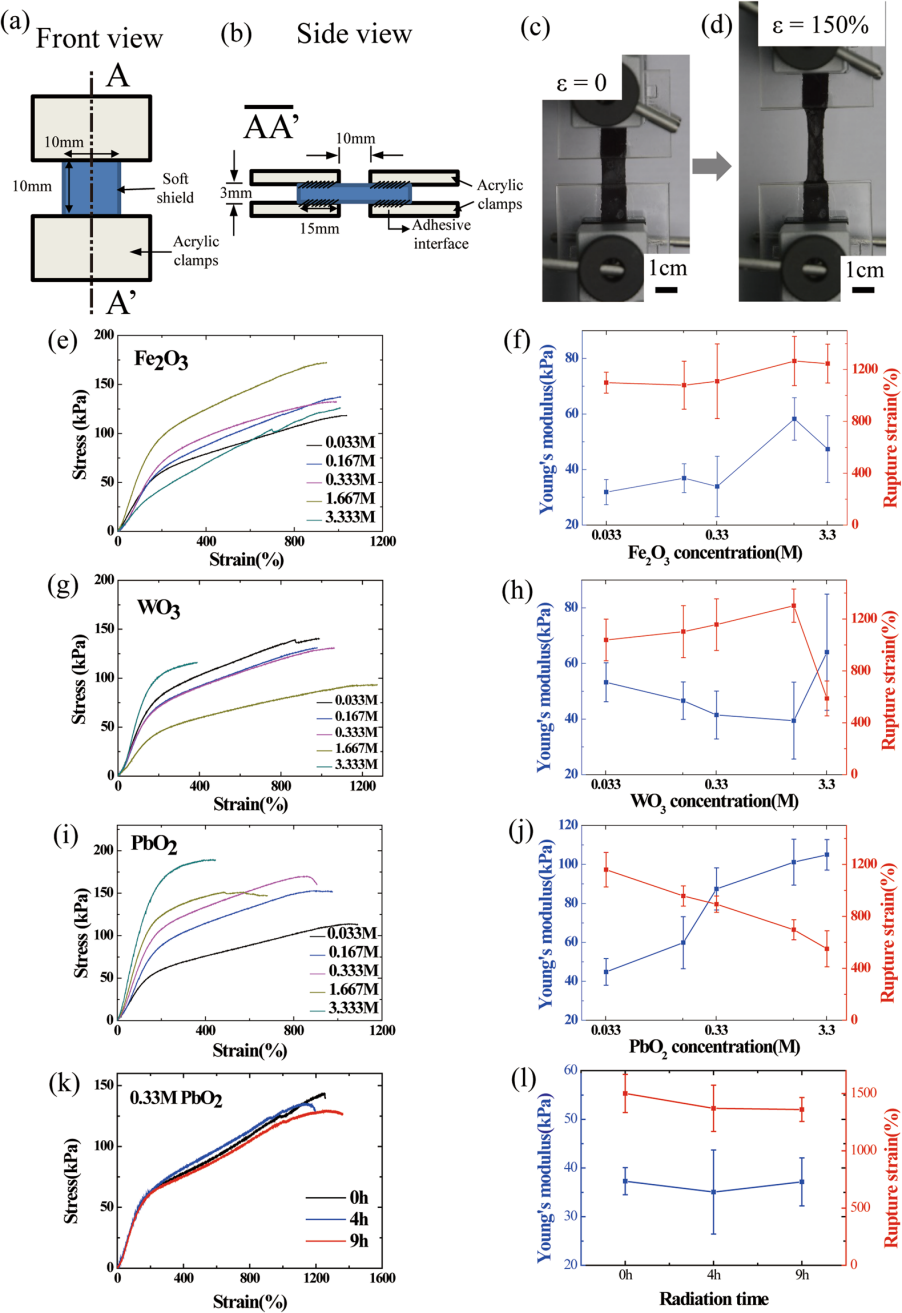
The soft shields are highly stretchable and compliant. (**a**)–(**b**) The geometry of tensile specimens. (**c**)–(**d**) Tensile test of the soft shields containing 3.33 M of lead oxide (PbO_2_) before and after stretching up to a 150% strain, respectively. (**e**), (**g**), and (**i**) Stress–strain curves for the soft shields with various amounts of the shield particles until the mechanical fracturing of each sample. (**f**), (**h**), and (**j**) The Young’s modulus and the rupture strain of the soft shields with various amounts of the shield particles. (**k**) Stress-strain curves for the soft shields containing 0.33 M of lead oxide with irradiated time. (**l**) The Young’s modulus and the rupture strain of the soft shield with irradiated γ-ray time. The soft shields are also stable in γ-rays.

A tensile test was performed at room temperature using the Instron 3343 tensile machine (Instron, U.S.A.) with a 50 N load cell to determine the mechanical properties of the soft shield for the γ-ray radiation. The specimen size was adjusted to 10.0 × 10.0 × 3 mm^3^, as shown in Fig. 3(a,b). Each soft shield was mounted on the tensile tester and then stretched until a mechanical rupturing occurred with a loading velocity of 6 mm/min. Figure 3(c,d) show the tensile testing of the soft shield containing 3.33 M of lead oxide before and after the stretching up to 150% strain, respectively.

The stress–strain curves of the soft shields with various metal-oxide concentrations are shown in Fig. 3(e,g, and i). Regarding the soft shield including iron oxide, the shape of the stress–strain curve is similar within small differences. It can be seen that as the amounts of the tungsten oxide and lead oxide particles was increased, the ductility of the soft shield was decreased. Figure 3(f,h, and j) show the Young’s modulus and the rupture strain of the soft shields with various shield-particle concentrations. As the soft shields contain particle quantities that are more than 3.33 M, the crosslink-formation of the soft shields becomes difficult due to the interaction between the crosslinking polymer and the metal-oxide particles. In the case of the soft shield containing iron oxide, the rupture strain is almost 1000%, and only a slight change is evident regarding the concentration of the shielding particles. In addition, a slight increase of the Young’s modulus was observed as the concentration of shielding particle was increased. However, for the soft shields containing WO_3_ and lead oxide, the rupture strain decreased as the concentration of the shielding particle was increased. Alternatively, the Young’s modulus was increased as the concentration of the shielding particle was increased. It is expected that with a greater inclusion of the shielding particles, the soft shields will become more stiff but less stretchable.

The soft shields containing 0.33 M of lead oxide were irradiated with γ-rays. In order to evaluate the stability of the soft shields to γ-ray, a tensile test was conducted before and after irradiation of the γ-ray. The γ-ray source was Cs-137 and the soft shields were irradiated for 4 hours and 9 hours. The stress-strain curves of the soft shields containing 0.33 M of lead oxide are shown in Fig. 3(k). The shape of the stress-strain curves before and after the γ-ray irradiation is similar. The Young’s modulus and rupture strain before and after irradiation of the γ-ray shows little difference within the error range as shown in Fig. 3(l). Therefore, the soft shields are stable in γ-rays.

**Figure 4:**
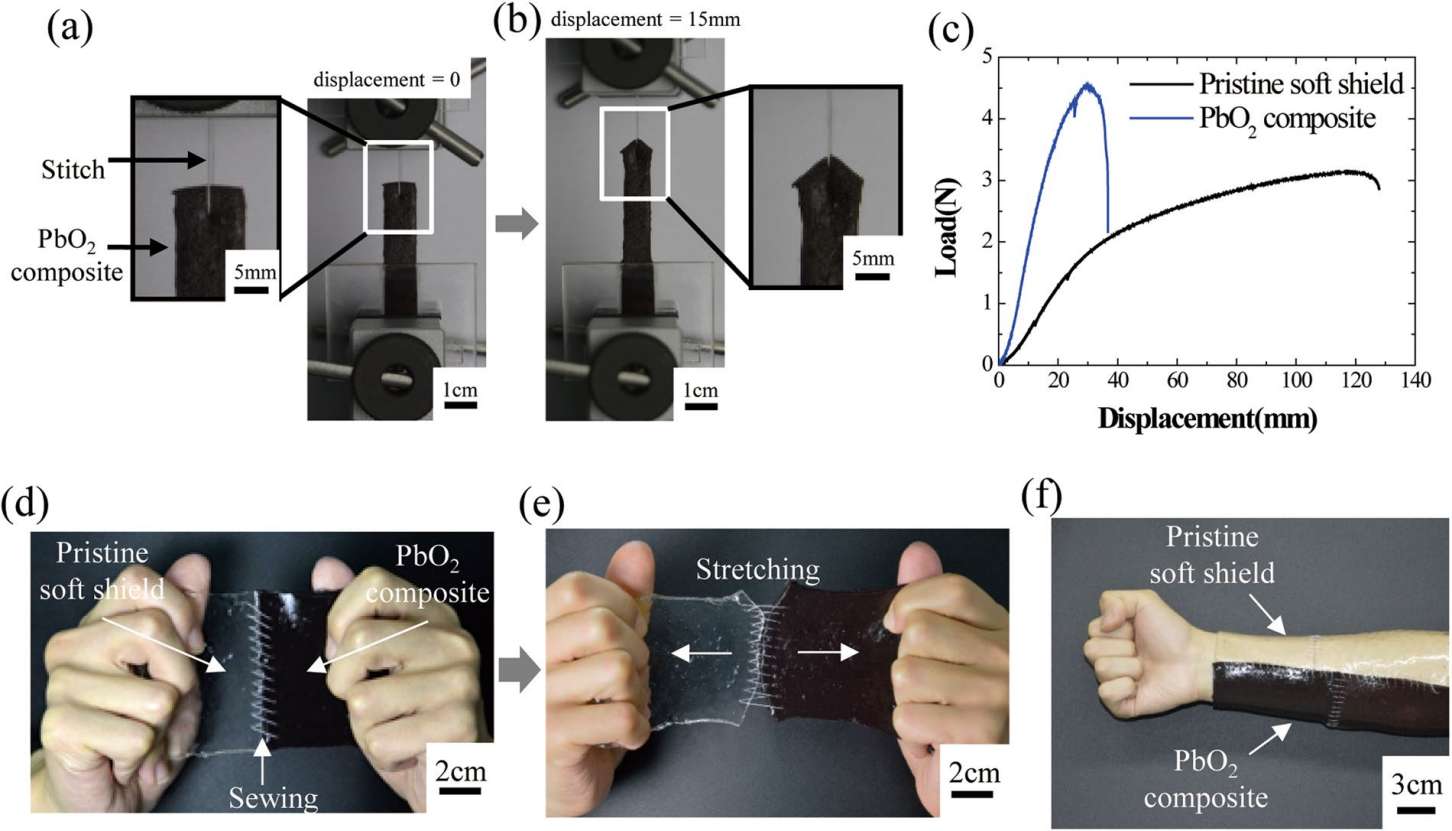
Sewable soft shields for the γ-ray radiation. (**a**)–(**b**) Stitch test of the soft shields containing 3.33 M of lead oxide (PbO_2_). (**c**) Load-displacement curves of the soft shields under stitch tests. A pristine soft shield and a soft shield with 3.33 M PbO_2_ were examined. (**d**)–(**e**) A pristine soft shield was connected to a shield with PbO_2_ by sewing. Both shields were kept intact after a stretching. (**f**) A wearable soft shield for the γ-ray radiation.

The soft shields are soft but their mechanical toughness is sufficient for sewing. A stitch test was performed with the soft shield, as shown in Fig. 4(a–c). The specimen size was adjusted to 10.0 × 25.0 × 3 mm^3^, and a 100-µm silk-fiber diameter was sewed at the middle, 5 mm lower than the top surface, as shown in Fig. 4(a,b). The stitch test for the sewed gel was performed with a loading rate of 6 mm/min. As shown in Fig. 4(a,b), the stitched specimen was mounted on the tensile machine and then stretched until a mechanical rupture occurred from the stitching site.

A load-displacement curve of the stitched soft shields is shown in Fig. 4(c). A pristine soft shield was ruptured at the 128 mm displacement, but the soft shield containing 3.33 M lead oxide was ruptured at the 37 mm displacement. But interestingly, the soft shield containing lead oxide was able to withstand a load that is 1.5 times higher than that of the pristine soft shield by only one stitch. Due to the excellent energy dissipation in the soft shields from the interpenetration of the covalently crosslinked polyacrylamide with the ionically crosslinked alginate, a sewing may mean that the shield can withstand high stress concentrations. The soft shield was sewn several times, as shown in Fig. 4(d), and it was stretched with considerable power, as shown in Fig. 4(e); therefore, it is possible that they could provide resilience without the occurrence of a rupture. As shown in Fig. 4(f), a wearable shield for the γ-ray radiation was simply made. The biocompatibility of the shield is expected to be high because the pristine hydrogel is biocompatible and the metal oxides that have been used in this work are nonreactive in a hydrogel matrix. Furthermore, it is expected that the shield will provide heat protection, since it contains a 51 wt% of water.

## Conclusion

A sewable soft shield for radiation was synthesized through an integration of hydrogels and metal oxides. Sewable soft shields with a shielding ability and a wearability are applicable in areas of the nuclear industry such as transportation, the storage of radioactive materials, and the protection of the human body following a radioactive accident. In the case of the soft shields containing 3.33 M of Fe_2_O_3_, WO_3_, and PbO_2_, the attenuation coefficients are 0.229, 0.251, and 0.284 cm^−1^, respectively, and they were stretched by more than 400% without the formation of a rupture. The stretchability and energy-dispersion ability of the fabricated soft shield are high so that sewing can be performed. If the soft shield contains greater amounts of metal-oxide particles or higher-atomic-number metals, the attenuation coefficient of the soft shield can be increased because the probability of the interaction between the γ-ray and the electron is increased. The attenuation coefficient can be calculated with the amount of the contained shielding material using analytic calculations, so the control of the attenuation coefficient and the mechanical properties of the soft shield can be achieved by adjusting the contained material and contents. Accordingly, the soft shield can be used as a wearable shield in a radioactive environment, enabling researchers to form a more comprehensive understanding of soft shields, thereby broadening the current soft-shield research and applications for radiation.

## References

[1] Brumfiel G (2013). Fukushima: Fallout of fear. Nature.

[2] Ten Hoeve JE, Jacobson MZ (2012). Worldwide health effects of the Fukushima Daiichi nuclear accident. Energy & Environmental Science.

[3] Siegbahn, K. Alpha-, beta-and gamma-ray spectroscopy. (Elsevier, 2012).

[4] Bushberg JT (2007). Nuclear/radiological terrorism: emergency department management of radiation casualties. The Journal of emergency medicine.

[5] Nelson, G. & Reilly, D. Gamma-ray interactions with matter. Passive Nondestructive Analysis of Nuclear Materials, Los Alamos National Laboratory, NUREG/CR-5550, LAUR-90-732, 27–42 (1991).

[6] Akkurt I, Akyildirim H, Mavi B, Kilincarslan S, Basyigit C (2010). Gamma-ray shielding properties of concrete including barite at different energies. Progress in Nuclear Energy.

[7] Yılmaz E (2011). Gamma ray and neutron shielding properties of some concrete materials. Annals of Nuclear Energy.

[8] Singh K, Singh N, Kaundal R, Singh K (2008). Gamma-ray shielding and structural properties of PbO–SiO 2 glasses. Nuclear Instruments and Methods in Physics Research Section B: Beam Interactions with Materials and Atoms.

[9] Harrison C (2008). Polyethylene/boron nitride composites for space radiation shielding. Journal of applied polymer science.

[10] Qin F, Brosseau C (2012). A review and analysis of microwave absorption in polymer composites filled with carbonaceous particles. Journal of applied physics.

[11] Bartczak Z, Argon A, Cohen R, Weinberg M (1999). Toughness mechanism in semi-crystalline polymer blends: II. High-density polyethylene toughened with calcium carbonate filler particles. Polymer.

[12] Allaoui A, Bai S, Cheng H-M, Bai J (2002). Mechanical and electrical properties of a MWNT/epoxy composite. Composites Science and Technology.

[13] Kim C-C, Lee H-H, Oh KH, Sun J-Y (2016). Highly stretchable, transparent ionic touch panel. Science.

[14] Keplinger C (2013). Stretchable, transparent, ionic conductors. Science.

[15] Lee YY (2016). A Strain-Insensitive Stretchable Electronic Conductor: PEDOT: PSS/Acrylamide Organogels. Advanced Materials.

[16] Annabi N (2016). Highly Elastic and Conductive Human-Based Protein Hybrid Hydrogels. Advanced Materials.

[17] Calvert P (2009). Hydrogels for soft machines. Advanced materials.

[18] Lake, G. & Thomas, A. in Proceedings of the Royal Society of London A: Mathematical, Physical and Engineering Sciences. 108–119 (The Royal Society).

[19] Gong JP, Katsuyama Y, Kurokawa T, Osada Y (2003). Double-network hydrogels with extremely high mechanical strength. Advanced Materials.

[20] Haraguchi K, Takehisa T (2002). Nanocomposite hydrogels: a unique organic-inorganic network structure with extraordinary mechanical, optical, and swelling/de-swelling properties. Advanced Materials.

[21] Ma J (2016). Highly Stretchable and Notch-Insensitive Hydrogel Based on Polyacrylamide and Milk Protein. ACS Applied Materials & Interfaces.

[22] Sun J-Y (2012). Highly stretchable and tough hydrogels. Nature.

[23] Ehmann, W. D. & Vance, D. E. Radiochemistry and nuclear methods of analysis. (1993).

[24] Hubbell J (1982). Photon mass attenuation and energy-absorption coefficients. The International Journal of Applied Radiation and Isotopes.

[25] Hubbell, J. H. & Seltzer, S. M. Tables of X-ray mass attenuation coefficients and mass energy-absorption coefficients 1 keV to 20 MeV for elements Z = 1 to 92 and 48 additional substances of dosimetric interest. (National Inst. of Standards and Technology-PL, Gaithersburg, MD (United States). Ionizing Radiation Div., 1995).

